# Application of Raman Spectroscopy and Micro‐Indentation to Micro‐Map the Path and Boundary of NaOCI‐Induced Dentine Collagen Changes in an Ex‐Vivo Root Canal Irrigation Model

**DOI:** 10.1002/cre2.70262

**Published:** 2025-12-15

**Authors:** Yuan Ng, Michele Recchia, Cristina Pereira, Graham Palmer, Laurent Bozec, Kishor Gulabivala

**Affiliations:** ^1^ Unit of Endodontology, Divisions of Restorative Dental Science, UCL Eastman Dental Institute University College London London UK; ^2^ Biomaterials & Tissue Engineering, UCL Eastman Dental Institute University College London London UK; ^3^ Faculty of Dentistry University of Toronto Toronto Canada

**Keywords:** collagen, dentine, immature teeth, micro‐indentation, Raman spectroscopy, sodium hypochlorite

## Abstract

**Objectives:**

To apply Raman spectroscopy and micro‐indentation to micro‐map the path and boundary of NaOCl‐induced dentine collagen changes in an ex‐vivo root canal irrigation model.

**Material and Methods:**

Root canals of extracted single‐rooted teeth were prepared and irrigated with NaOCl or saline. Four teeth (NaOCl = 3; saline = 1) embedded in epoxy resin and sectioned transversely into discs were Raman‐analyzed on coronal surfaces from *inter‐* and *intra‐tubular* dentine in 4 quadrants over 12, 24, and 48 min‐acquisition times. Eight additional teeth stratified by root maturity, irrigated with NaOCl (*n* = 7) or saline (*n* = 1), sectioned transversely and then embedded, were Raman‐analyzed on apical surfaces at 18 equidistant (50 μm) points/quadrant for Amide bands. Micro‐indentation of the corresponding facing sectioned surface was correlated with Amide band changes. Generalized linear and non‐linear regression models were used for data analysis.

**Results:**

Spectral quality at 24/48 min was similar and better than at 12 min. Inter‐tubular but not intra‐tubular spectra were masked by fluorescence. Spectral features near the canal lumen ( < 500 μm) showed more significant collagen alteration and varied by tooth/quadrant but decreasingly towards the cemento‐dentinal junction (CDJ) without a clear boundary. Significant (*p* < 0.0001) changes in Amide I/III bands up to 300 μm from the canal and were accompanied by deeper corresponding indentations upto 200 μm. Canal instrumentation had a significant (*p* < 0.0001) effect on both Amide‐I and Amide‐III bands.

**Conclusions:**

NaOCl altered dentinal collagen and reduced microhardness but varied with quadrants/teeth, without a definable boundary; collagen changes were obvious within 300 μm of the canal and microhardness changes within 200 μm but evident to a decreasing extent up to the CDJ.

## 1 Introduction

Sodium hypochlorite used for root canal debridement to control the microbial infection that causes periapical disease is known to weaken dentine (Grigoratos et al. 2001; Rajasingham et al. 2010; Sim et al. 2001) through chemical changes in its organic phase (Driscoll et al. 2002; Pascon et al. 2012; Ramírez‐Bommer et al. 2018) and is deemed an unintentional risk of root canal treatment. However, the full extent of the dentinal structure alteration that could determine the long‐term risk of tooth survival has not yet been characterized. Therefore, a key knowledge component for rational clinical decision‐making is missing.

Fourier transform infrared (FTIR) spectroscopy has been successfully employed to assess dentine collagen changes caused by NaOCl (Amarie et al. 2012; Browne et al. 2020; Di Renzo et al. 2001a, 2001b; Morgan et al. 2019; Ramírez‐Bommer et al. 2018; Tartari et al. 2016). NaOCl dissolves, disintegrates, or denatures dentinal collagen in the dentine matrix (Hu et al. 2010; Tartari et al. 2016; Zhang, Kim, et al. 2010; Zhang, Tay, et al. 2010). The overall extent of *tooth weakening* caused by such collagen changes during and following root canal irrigation is likely to be dictated by the breadth and depth of affected dentine relative to the remaining bulk of unaffected dentine (Rajasingham et al. 2010). The *depth* of effect of alternate exposure of pulverized dentine to NaOCl and EDTA was *estimated* using FTIR (Ramírez‐Bommer et al. 2018) but the model was unsuitable for providing in‐situ insight. In pulverized dentine, the estimated depth of hypochlorite reaction was found to be 16 ± 13 µm after 10 min, whilst the depth of EDTA reaction increased with duration of exposure (19 ± 12 µm by 10 min, 27 ± 13 µm by 60 min, and 89 ± 43 µm by 24 h) and by pre‐treatment with NaOCl (62 ± 28 µm by 10 min). NaOCl reduced the collagen content in dentine within the first 4 min, plateauing thereafter, whilst EDTA continuously reduced phosphate content over 24 h, exposing more collagen in the process (Ramírez‐Bommer et al. 2018).

Morgan et al. (2019) evaluated the extent of dentine collagen alteration through root canal irrigation in‐situ by assessing changes in IR absorption frequencies using an ex‐vivo experimental model. The effect of NaOCl on dentinal collagen could be traced to at least 0.5 mm into the dentine from the root canal wall, although the relatively large probe size limited the test resolution. Using an identical model, Browne et al. (2020) explored the extent of the effect of NaOCl penetration using immature and mature teeth, with or without periodontal disease affliction, to assess the impact of patent, smear‐occluded, or sclerosed dentinal tubules. Both root maturity and irrigation protocol influenced the ability of NaOCl to alter dentinal collagen up to 0.5 mm deep from the canal lumen surface; the extent of effect assessment was, again, limited by the test method resolution as in the previous study.

Raman spectroscopy offers an alternative in‐situ mapping technique for depicting the spatial distribution of organic and inorganic compounds in dentine with much better spatial resolution at the smaller scale of 1 μm (Casciani et al. 1979; de Mul et al. 1986; Garidel and Boese 2007; Nelson and Featherstone 1982; Pelletier and Pelletier 1999; Tramini et al. 2000).

The severity of chemical changes may be correlated with the hardness of dentine and used as an adjunctive measure (Saleh and Ettman 1999). Dental studies have adopted the Vickers test (Mollica et al. 2012), nano‐indentation (Hayashi et al. 2012) and micro‐indentation (reference point indentation [RPI]) for hardness measurement. The Vickers test applies 0.01–981 N on a single pyramidal probe in one application, but its spatial resolution is poor due to a larger probe size. Conventional nano‐indenters measure a length scale of the order of 10–9 nm with a force range of 1.0–500 mN. The probe size is microscopic and allows a high density of probing points with the drawback of extensive sample preparation (flattening, cleaning, dehydration, smoothening, polishing) that may distort the results (Hayashi et al. 2012).

Micro‐indentation uses two probes: a reference and a test probe. The reference probe ensures a firm position on the sample, allowing the test probe to indent over a series of cycles. It measures both probe penetration (10–6 μm) and corresponding force (2.0–10.0 N) and allows ex‐vivo sampling (Diez‐Perez et al. 2010). The indentation cycle has three phases: the indenting phase gives the initial depth of penetration, followed by the depth during the holding phase and the end of the cycle gives the “Total Indentation Distance.”

The aim of this study was to develop and apply a protocol for more precisely mapping the path and depth of NaOCl's effect on collagen changes in the root dentine of mature and immature teeth using Raman Spectroscopy and micro‐indentation in an established ex‐vivo root canal irrigation model.

## 2 Methodology

### 2.1 Collection, Storage and Preparation of Sample Teeth

**Figure 1:**
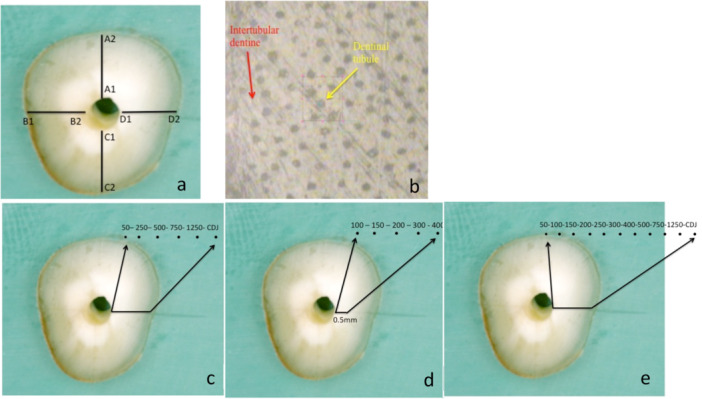
Dentine disc scored with 4 lines designated A (buccal), B (mesial), C (lingual), D (distal), b—Raman scanning points in intra‐tubular (yellow arrow) and inter‐tubular (red arrow) dentine, c, d, e—points of Raman scanning for various experiments. CDJ = cemento‐dentinal junction.

Approval for the use of extracted teeth was granted from the UCL Eastman Biobank (Study number: 1301). Informed consent was obtained for all the extracted maxillary central incisor teeth donated to the UCL Eastman Biobank. The UCL Eastman Biobank received favorable ethics opinions from Yorkshire and The Humber—Leeds East Research Ethics Committee (REC reference number: 22/YH/0146). The design and report of this study followed the “Preferred Reporting Items for Laboratory studies in Endodontology (PRILE) 2021 guidelines” (Supporting Information S1). The teeth were decontaminated in 0.1% Thymol (BDH Laboratory Supplies, Poole, England) and stored in 70% ethanol (BDH Laboratory Supplies, Poole, England) with deionized water at 4°C until processing. From these, intact crack‐free and caries‐free single‐rooted teeth were selected for two sets of experiments (*n* = 12: T1–4 and T5–12). Four teeth, T1–4 (T1–3 = test samples; T4 = control sample) were used for Raman spectroscopy protocol development, data were collected at four points along each line drawn in four quadrants giving 16 data points per tooth, with a total of 48 data points from the test teeth and 16 data points from the control tooth (Figure 1). For protocol development, a sample size of 3 teeth with 48 data points were deemed sufficient to observe consistency of spectra quality. For the main experiment, seven test teeth (T5–11) with 18 equidistant points at each quadrant to give a total of 504 data points; one tooth (T12) was used as control (Figure 1). Due to the lack of previous data for formal sample size estimation, the sample size of seven teeth was based on a previous study using FTIR (Browne et al. 2020). They had also used seven teeth per group for between group comparison.

#### 2.1.1 Sample and Root Canal Preparation

The crowns of all teeth were ground flat incisally to standardize the reference point and canal length to 19 mm. After pulpal access, canal lengths were determined by advancing a file to the apical terminus. The canals were mechanically prepared to 0.5 mm short of the terminus using a combination of Flexo‐files and rotary ProTaper instruments (Dentsply‐Sirona, Charlotte, NC, USA), to a size and taper corresponding to Protaper F3. Deionized water was used as the irrigant between each instrument, maintaining teeth moist with soaked gauze during the process.

The apices of samples *T1–4* were then sealed with composite resin (XRU Herculite, Kerr Corporation, Orange, CA, USA) and the teeth embedded in clear epoxy resin (Specifix 40, Struers Ltd. Solihull, West Midlands, UK) using a cylindrical mould. These samples were used to determine the optimal Raman spectrum acquisition protocol to be used on samples *T5–12*.

The tooth maturity and degree of canal instrumentation were determined for samples *T5–12* to account for their potential confounding effect on the measured outcomes. Before canal preparation, perpendicular periapical radiographs (mesio‐distal and bucco‐lingual) of these samples (*T5–12*) were used to measure the thickness of the root apically, relative to the canal width to quantify tooth maturity. Image‐Pro Plus (Media Cybernetics Inc. Rockville, MD, USA) was used to draw a straight‐line parallel to the root axis and perpendicular to the apical‐most margin of the foramen, with 4 intersecting perpendicular lines at 0.25 mm, 0.5 mm, 0.75 mm, and 1.0 mm from the foramen. The thickness of the root and width of the canal were measured at the intersection lines using a digital calliper, allowing the average apical root thickness (RT) and apical root canal width (CW) to be calculated to derive a “maturity index” (MI = RT/CW).

The samples *T5–12* then had a square slot (2.5 × 2.5 × 3 mm) cut in the root face perpendicular to the coronal plane with a handpiece‐driven bur. An impression (V1) was taken of the canal with light‐body silicone impression material (Aquasil Ultra LV/XLV Smart Wetting Regular Set, Dentsply Sirona) using a Hedström file (size 45) to the standard length as the central core to provide a handle and aid its removal. After canal preparation, a second impression (V2) of the canal was taken using the coronal slot for relocation. The impressions (V1, V2) allowed determination of the extent of instrumentation. After separating the Hedström file handle from the impressions with a rotary disk, the weights of the impressions were determined on a precise scale (AG204 Delta Range Balance, Mettler, Toledo, UK), and Archimedes principle was used to calculate their volumes. The degree of instrumentation was determined by subtracting V1 from V2. The roots of samples T5–12 were then embedded in silicone putty (Speedex, Coltene/Whaledent Ltd, West Sussex, UK) to protect the external surface against the irrigant and to allow transverse splitting of the teeth for spectroscopy and micro‐indentation tests on the corresponding opposing split surfaces.

#### 2.1.2 Root Canal Irrigation

The *prepared* canals of 10 teeth (T1–3; T5–11) were irrigated with 5% NaOCl (Merck Chemicals, Nottingham, UK, confirmed by iodometric titration) according to a standardized protocol; whilst 2 teeth (T4, T12) were assigned as controls, were irrigated with saline using an identical protocol. Irrigant was delivered using a total 3 mL Monoject syringe (Tyco Healthcare, Gosport, UK) through a 27‐gauge Maxi‐Probe needle (Dentsply Rinn, Elgin, IL, USA), the tip of which was placed 7 mm from the coronal surface. This pre‐set level was used to control irrigant delivery over 1 min using a push‐pull motion of amplitude 4 mm *above* the pre‐set position judged by the needle tip as a reference. The canals were then left exposed to the delivered irrigant for 4 min. This process was repeated four more times, resulting in a total exposure time of 25 min to 15 mL in 4 teeth (T1–3 NaOCl, T4 control saline). Whilst, in 7 teeth (T5–11 NaOCl, T12 control saline), 6 mL of irrigant was delivered over 1 min, and the solution was left in the canal for a further 4 min. Five such irrigation cycles delivered a total of 30 mL over 25 min. The effect of NaOCl was quenched by washing the canal with 6 mL of deionized water over 1 min.

### 2.2 Application of Raman Spectroscopy for Assessing Collagen Changes in Dentine Ex‐Vivo

#### 2.2.1 Preparation of Dentine Discs for Raman Analysis

Following irrigation, the resin cylinders for *T1–4* were sectioned transversely at 6 and 9 mm below the incisal reference using a diamond microtome (Leica Model 1600, Leica, Wetzlar, Germany) to yield 3 mm thick discs displaying a transverse cross‐section of the root and canal.

The coronal portions of the, yet un‐embedded *T5–12* were separated from the root with a *transverse split* using pre‐grooving with a diamond disc to avoid grinding the future sampling sites. Scalpel blades inserted into the groove on opposite sides enabled a split with a gentle tap using a 50‐g hammer. The coronal and apical portions were then embedded in cylindrical moulds in clear epoxy resin (Specifix 40, Struers Ltd. Solihull, West Midlands, UK). The apical root portion was stored in 0.1% Thymol for micro‐indentation.

The reinserted V1 impression was securely relocated by the square hole, allowing the apically exposed root impression to be cut flush with a scalpel blade. An image (Nikon D90/macro‐105 mm) of the apical root face was captured alongside a correctly aligned ruler. The extent of canal instrumentation (EI) was determined using a magnified digital image as the linear distance from the impression margin to the canal wall (mm) in every quadrant per tooth, using Image‐Pro Plus software (Media Cybernetics Inc. Rockville, MD USA).

The coronal surfaces of discs *T1–4* and the apical surfaces of discs *T5–12* were polished with increasingly fine‐grade abrasive disks (500, 1200, 2400 μm grit) (Struers Ltd., Solihull, West Midlands, UK) at 300 RPM for 1 min under water coolant. The polishing was finished with diamond pastes of 9, 3, and 1 µm (Diapro, Struers Ltd. Solihull, West Midlands, UK) for 5.5, 4.5, and 1.5 min, respectively.

#### 2.2.2 Raman Spectroscopy

Four lines were scored with a scalpel blade on the polished surface from the canal lumen to the cemento‐dentinal junction (CDJ) (Figure 1a), designated: A (buccal), B (mesial), C (palatal), and D (distal). The lengths of the lines measured under an operating microscope (×6 magnification, Urban microscope, DP Medical Systems, Chessington, Surrey, UK) gave the dentine thickness on the respective sides. The scored disc was ultrasonicated for 30 min in deionized water, dried and treated with 1 mL of 17% EDTA solution for 30 s (Pulpdent, Watertown, MA, USA), then washed with copious amounts of deionized water and dried with absorbent towels.

The dentine surface was analyzed using a Raman microscope and spectra were recorded on Lab‐Ram 300 spectrophotometer (Jobin Yvon, Horiba, France) using monochromatic radiation emitted by He‐Ne laser with a maximum nominal power of 20 mW at a wavelength of 632.8 nm. The monochromator was equipped with a grating of 950 grooves/mm, allowing a maximum spectral wavenumber resolution of 1 cm^−1^ for samples T1–4. The laser light was focused on the sample surface using a 50× optical objective (Olympus MPLAN 50×/0.75) on an excitation area of about 5 μm diameter (aperture).

#### 2.2.3 Determination of Optimal Duration and Location for Spectrum Acquisition

Spectra from inter‐ and intra‐tubular dentine were evaluated for interference with background dentine fluorescence (Figure 1b). One sample (T1) was used to record 3 *inter‐tubular* spectra 500 μm from the *canal wall* (D1), using three different acquisition times (12, 24, 48 min). Another 3 *inter‐tubular* dentine spectra were recorded in the same quadrant, 500 μm from the CDJ (D2), and the same acquisition times. Two further spectra were also taken from *intra‐tubular* dentine at D1 and D2 (Figure 1a), respectively, with an acquisition time of 24 min. The spectra were processed and compared, and the signal‐to‐noise ratio (SNR) for the Amide I band at locations D1, and D2 was analyzed by the duration of acquisition.

Two samples (T2, T3) were used to obtain 2 *intra‐tubular* dentine spectra (500 μm from the canal wall, 500 μm from CDJ) from each of the four quadrants (A–D) per tooth. The control sample T4 was used to obtain only 2 intra‐tubular dentine spectra from quadrant A. Spectra were processed and analyzed at Amide I (1667 cm^−1^) and Amide III (1243 cm^−1^) bands.

#### 2.2.4 Determination of the Boundary of NaOCI‐Induced Changes in Dentine Collagen

Initially, intra‐tubular dentine spectra with 24‐min acquisition times were taken at 6 points from each quadrant of two samples (T2, T3). The 6 points were distributed along the scored line at 50, 250, 500, 750, and 1250 μm from the canal wall and at a point close to the CDJ (Figure 1c). For the control (T4), the six spectra from the respective points were only obtained from quadrant A. A further five spectra were taken within the first 500 μm from the canal wall of T2 and T3 (quadrants A–D) and T4 (quadrant A) with higher spatial resolution at 100, 150, 200, 300, and 400 μm from the canal wall (Figure 1d). The spectra were processed and analyzed as before and with OriginLab software. The data for T2 (44 data points from all four quadrants) and T4 (11 data points from one quadrant) were separately pooled to analyze the change in Amide I band height over the distance from the canal wall using non‐linear regression (OriginLab).

For samples *T5–12*, the laser was focused utilizing the ×50 magnification objective (Olympus MPLAN 50×/0.75); diffraction grating of 1800 gr/mm and 1 × 1 μm detecting area were chosen to ensure the highest achievable resolution. Spectra were recorded over a wavelength of 1350 cm^−1^ with an acquisition time of 24 min (exposure time of 5 s, and accumulation number of 256). For every sample treated with NaOCl (*T5–11*), Raman spectroscopy was performed on intra‐tubular dentine at 18 equidistant points (50 μm) in each quadrant from the canal wall towards the CDJ with an additional point taken 50 μm from the CDJ (Figure 1e). For T12 (deionized water), the spectroscopy was performed on 6 equidistant (150 μm) points in the buccal quadrant.

Raman spectra were acquired in single‐point acquisition mode rather than mapping mode. Each point represented a discrete location along a predefined transect from the canal lumen to the CDJ, with 18 equidistant points per quadrant. The laser spot size was approximately 1 × 1 µm, and the acquisition area per point corresponded to a single pixel. Although mapping mode could have provided spatially continuous data, the extended acquisition time required for high‐resolution mapping across multiple quadrants and depths was not feasible within the constraints of the study. Instead, a high‐density point sampling strategy was adopted to balance spatial resolution with spectral quality and experimental throughput. The 24‐min acquisition time per point was determined through preliminary optimization, which showed that shorter durations yielded suboptimal SNR, particularly for the Amide I and III bands.

#### 2.2.5 Data Analysis

Acquired spectra were analyzed using LabSpec5 Raman software (Version 5.78.24; Jobin Yvon, Horiba, France). The spectra were baseline corrected, 8‐points smoothed, and normalized on the carbonate (CO_2_^−3^) bands 1072 cm^−1^ and overlaid for direct visual comparison or using the waterfall analysis function of OriginPro9 (OriginLab) to display the curves between 1050 cm^−1^ and 1750 cm^−1^, to capture the collagen fingerprints (Amide I—1667 cm^−1^; Amide III—1243 cm^−1^). The maxima absorbances for Amide I (1667 cm^−1^), Amide III (1243 cm^−1^), and carbonate (1072 cm^−1^) bands were recorded. The Amide III band was assessed for visual changes in its band doublet slope, width, and height. The spectra were all offset from 0 to 1700 cm^−1^ before recording the absorbance. The data were exported to Microsoft Excel (Microsoft Inc., Redmond, WA, USA) software.

The recorded spectra were also qualitatively rated because degraded collagen yielded sub‐optimal spectra; the rating assessed the height of Amide bands compared to the standardized carbonate band 1072 cm^−1^ and as a function of accessory abnormal bands. For this purpose, each spectrum (1050 cm^−1^ to 1750 cm^−1^) was divided into three areas, corresponding to Amide III (1615 cm^−1^ to −1719 cm^−1^), Amide I (1215 cm^−1^ to −1302 cm^−1^) and the 1450 cm^−1^ band. Each area was rated 1 (optimal) to 3 (unacceptable) (Table 1), and the entire spectrum was allocated a general score. Only spectra with QI ≤ 6 were included in the integrated area analysis of Amide III and I bands. The overall general quality score did not allow spectra with one of its portions rated 3 to be included ([3 + 1 + 1] × 2 = 10) but allowed spectra with each portion rated 2 to be accepted for the integrated Amide band area analysis ([2 + 2 + 2] × 1 = 6). The spectra were used to calculate the integrated area of the Amide I and III. The degree of root maturity and extent of instrumentation were used as dependent variables, and a linear regression analysis (STATA software, version 17, STATA Corporation, College Station, Texas, USA) was performed to evaluate their potential influence on Amide areas and micro‐hardness as outcome variables.

**Table 1:** Criteria for scoring quality of spectra.

Score	Criteria
1	Peaks between 25% and 75% of height of control peak at 1072 cm^−1^
2	Amide accessory peak 25%–75% of height of control peak at 1072 cm^−1^
3	Amide accessory peak higher than the main amide pick and/or with amide band height below the 25% or above the 75% of the control at 1072 cm^−1^
Overall general quality	Score 1 given to a spectrum with each of its portions rated 1/2. Score 2 given to spectrum with one portion rated score 3 (unacceptable). Score 3 given to spectrum not resembling the collagen spectrum in any part.
Overall score QI =	Quality of Amide III + quality of Amide I + quality of peak at 1450 × overall general quality.

### 2.3 Reference‐Point Indentation to Investigate the Depth of Effect of NaOCI on Dentine

A BioDent Hfc was used for cyclic micro‐indentation (Active life Scientific, Santa Barbara, CA, USA). The root halves intended for the micro‐indentation tests were adapted to fit the Bio‐dent holding tray and reproducibly secured to the micro‐metrically controlled X‐Y table, allowing the samples to be moved in 40 μm increments.

The embedded teeth were kept in 0.1% Thymol until placed on the BioDent table and covered with moist gauze. The reference probe was placed in contact with the sample, and the reference force was increased until its value reached the Reference Force range (12.7 N–13.2 N). The amplitude of movement of the probe (Touchdown Distance) was adjusted to approximately 150–140 μm according to the acceptable range for the chosen indenting force of 10.0 N (50–250 μm). Every test resulted from 20 indentation cycles with a frequency of 2 cycles per second (2 Hz). Every tooth received a variable number of tests for each quadrant depending on the thickness of its dentinal walls. The probe was moved along the surface of the section from the CDJ towards the canal lumen following a pattern of advancement of 200 μm increments with concomitant alternating excursions of ±100 μm perpendicular to the direction of advancement. This oblique advancement ensured the entire quadrant was tested (as the probe indents on average 200 μm in dentine), allowing a safe distance between the indented areas of almost 100 μm.

## 3 Results

### 3.1 Raman Spectroscopy Protocol for Assessing Alterations in Dentine Collagen

#### 3.1.1 Optimal Duration of Spectrum Acquisition

The longer acquisition times gave better SNR (Table 2) with more defined bands. Spectra obtained closer to the CDJ (D2) yielded better SNR than those near the canal (D1). All subsequent spectra were acquired over 24 min.

**Table 2:** Signal‐to‐noise ratios for Amide I band of T1 (locations D1 and D2) by duration of acquisition.

	SNR (D1)	SNR (D2)
12 min	28/8 = 3.5	62/7 = 8.8
24 min	50/5 = 10	50/3 = 16.6
48 min	50/5 = 10	39/2 = 19.5

*Note:* D1 = 500 μm from canal wall; D2 = 500 μm from CDJ.

#### 3.1.2 Raman Spectra From Inter‐ and Intra‐Tubular Dentine

Fluorescence emission, devoid of the “collagen fingerprint” bands (Amide I and Amide III bands) as well as the carbonate band, tended to mask the Raman spectra from inter‐tubular dentine because of its greater intensity. In contrast, spectra acquired from intra‐tubular dentine at 1250 μm from the canal wall (T2) consistently gave characteristic Raman dentine band patterns, therefore, all subsequent Raman spectra were acquired from within dentinal tubules.

### 3.2 Effect of NaOCI Irrigation on Dentine Assessed by Raman Spectroscopy on T1–4

#### 3.2.1 Comparison of Dentine Collagen Spectra Taken Adjacent to the Canal Wall Versus the CDJ

**Figure 2:**
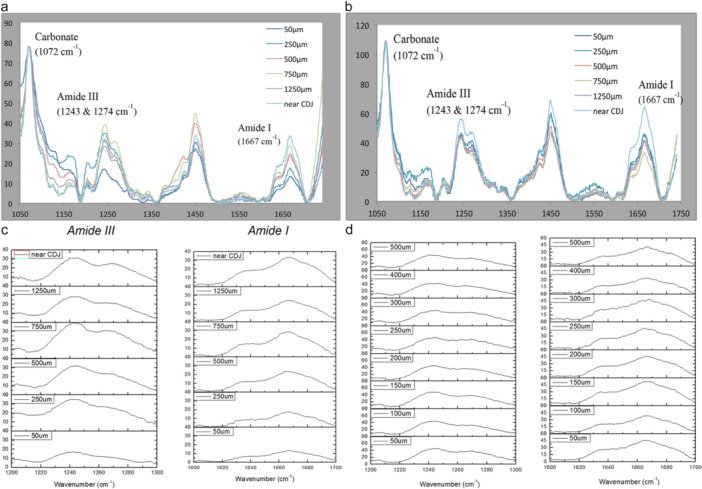
a Six overlaid Raman spectra comparing the Amide I and Amide III peaks for the collagen in sample T2 (quadrant A) at various intervals from CDJ to the canal wall. b Six overlaid Raman spectra comparing Amide I and Amide III peaks for collagen in control tooth (sample T4), quadrant A, at various intervals from CDJ to the canal lumen. c Raman spectra of T2 comparing Amide III and Amide I peaks at the six location points. d Raman spectra for Amide I (right) and Amide III (left) of T4 (control) detailing 500 μm from the canal lumen.

Raman spectra obtained from the four quadrants (A, B, C, D) of the NaOCl‐irrigated T2 (Figure 2a) and T3 (*similar to T2 spectra*) at the CDJ demonstrated higher Amide I band intensity (1667 cm^−1^) and the presence of the doublet slope in the Amide III band (1243 cm^−1^) compared with spectra obtained near the canal wall, which showed flattening of the doublet slope in the Amide III band. Changes in the spectral features included a broadening of the band accompanied by a loss of shoulder contour of the Amide III band and finally a decrease in intensity of the Amide I band compared with the canal wall and CDJ spectra after NaOCl irrigation. These changes were consistent with denatured collagen at 500 μm from the canal wall. Spectra obtained from T4 (saline‐irrigated control—Figure 2b) showed no obvious differences in the Amide I band intensity, or in the Amide III doublet slope or width at various intervals from the canal to the CDJ.

### 3.3 Estimation of the Boundary of NaOCI‐Induced Changes in Dentine Collagen

#### 3.3.1 Spectra Acquired From Intra‐Tubular Dentine at 6 Points Between the Canal Wall and the CDJ

The spectra obtained at various distances from the canal wall were overlaid following baseline correction and normalization on the carbonate band (1072 cm^−1^). Obvious changes were evident in the spectra from the first 2 locations (50 μm and 250 μm from the canal wall) for both Amide I and Amide III absorbance bands (Figure 2a). The Amide I band intensity was reduced in spectra closer to the canal wall. The Amide III contours also changed with the loss of the doublet slope and broadening of the bandwidth in the spectra closer to the CDJ. There were always combined or isolated changes in the Amide I and Amide III bands, characterizing disordering and denaturation of the collagen in the first 500 μm from the canal lumen in all samples irrigated with NaOCl.

The spectra from the control T4 (Figure 2b) showed no obvious difference in spectral appearance for Amide I and Amide III bands at various distances from the CDJ towards the canal wall. OriginLab plots confirmed that Amide III doublets remained at 50 μm from the canal lumen and there was no broadening of the bands. In the Amide I band, there was a slight decrease in height from CDJ to 1250 μm, and from then, it was maintained towards the canal wall.

To aid interpretation, the data were exported to OriginLab and rescaled to show wavenumbers from 1200 cm^−1^ to 1300 cm^−1^ and from 1600 cm^−1^ to 1700 cm^−1^ (Figure 2c). These confirmed band reduction of Amide I and loss of band shoulder of Amide III. The changes were more noticeable from 500 μm towards the canal wall. At 750 μm or further from the canal wall, the doublet Amide III spectrum was maintained, and Amide I bands were seen at similar heights. Data from T1, T2, and T3 were consistent.

#### 3.3.2 Spectra Acquired From Intra‐Tubular Dentine at 5 Points Within 500 μm From the Canal Wall

The further acquisition points between the canal wall and at 500 μm from the canal wall showed at higher spatial resolution (100 μm), the effect of NaOCl on dentinal collagen at points closer to the canal lumen. In contrast, the spectra obtained from various distances within the 500 μm boundary from the canal wall of T4 (control) all looked similar. There was no obvious difference in the broadness or contour of both Amide III and Amide I bands.

Data obtained from 50 μm to 500 μm from the canal wall were combined to produce a more detailed Amide I and Amide III spectral analysis of the entire 500 μm region close to the canal wall. The data were exported to OriginLab. These showed concurrent changes in Amide I (band intensity reduction) and Amide III (loss of doublet slope), more visible at 300 μm or closer to the canal wall. In T4 (saline control), the Amide III doublets were maintained from the CDJ to 50 μm from the canal wall, as were the band heights of Amide I, indicating that the collagen had not been affected.

**Figure 3:**
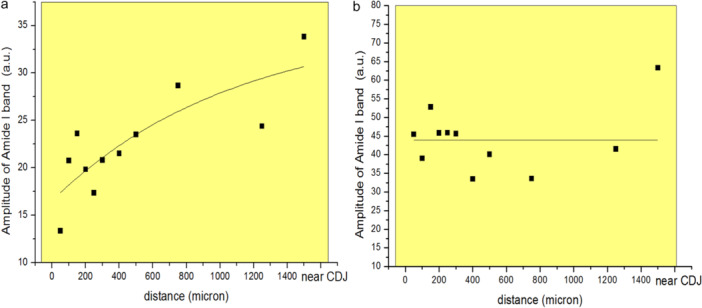
a Combined Raman spectra peak heights for Amide I in T2 (NaOCl) against distance from the canal lumen. b Combined Raman spectra peak heights for Amide I in T4 (control) against distance from the canal lumen.

The T2/T4 data obtained from 50 μm up to the CDJ (11 location points) were pooled for further analysis. The amplitudes of the Amide I band were plotted against the distance from the canal wall using OriginLab, and non‐linear regression analyses were performed (Figure 3). For T2, the best non‐linear regression line revealed that the slope of changed Amide I band amplitude increased with distance away from the canal wall (Figure 3a), while the best‐fit regression line for T4 revealed no apparent change in amplitude of Amide I over increasing distance from the canal wall (Figure 3b). Additionally, the amplitudes of the Amide I band in T2 were lower than those in T4. This could be due to reduced collagen signature relative to the carbonate in T2 compared with T4 because of the effects of NaOCl irrigation.

### 3.4 Effect of NaOCI Irrigation on Dentine Assessed by Raman Spectroscopy on T5–12

#### 3.4.1 Tooth Characteristics

The sample teeth were evenly distributed by state of maturity (Table 3). The mean canal volume of the teeth following preparation was 0.049 cm^3^ (95% CI: 0.048, 0.050) with narrow confidence intervals; as a result, “volume of canal” was not included in the statistical analysis. The mean extent of dentine removal through instrumentation was 0.048 mm (95% CI: 0.040, 0.056). The thickness of dentine from canal lumen to the CDJ ranged from 1200 μm to 2350 μm with an average of 1771 μm (95% CI: 1248, 2294).

**Table 3:** Frequency distribution of tooth maturity.

Maturity	Number of teeth
X ≤ 1.9	0
2. > X < 2.9	2
3 > X < 3.9	1
4 > X < 4.9	2
5 > X < 5.9	1
6 > X < 6.9	1
X ~ 11	1

#### 3.4.2 Qualitative Raman Spectrum Analyses

The plots’ x‐axes were rescaled to show wavenumbers for the Amide I (1615–1719 cm^−1^) and Amide III (1215–1302 cm^−1^) bands. Spectra from the saline‐irrigated tooth showed no obvious differences in the Amide I band intensity (1667 cm^−1^) and the double slope of Amide III (1243 cm^−1^) at the different sampling locations.

**Figure 4:**
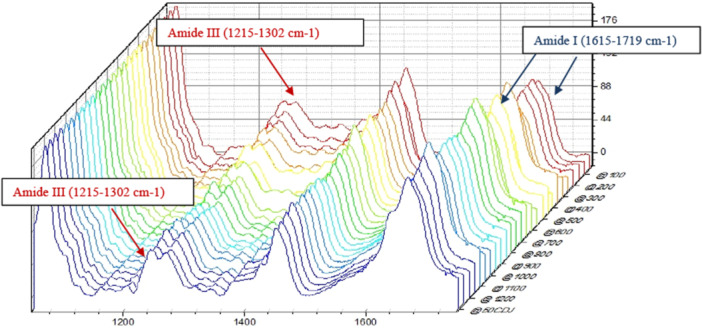
Example of waterfall plot of Raman spectra acquired from one single quadrant T2 D.

Spectra from NaOCl‐irrigated teeth acquired close to the canal lumen (Figure 4, Figure S2) showed a homogenous trend of loss of shoulder contour (double slope) on the Amide III band (red arrows), as well as a decrease in band intensity for the Amide I band (blue arrows) near the canal lumen. In every tested tooth and within the same tooth in each quadrant, the loss of contour and the height of the bands showed different trends, making localization of a distinct boundary effect complex per tooth or as an average trend.

**Figure 5:**
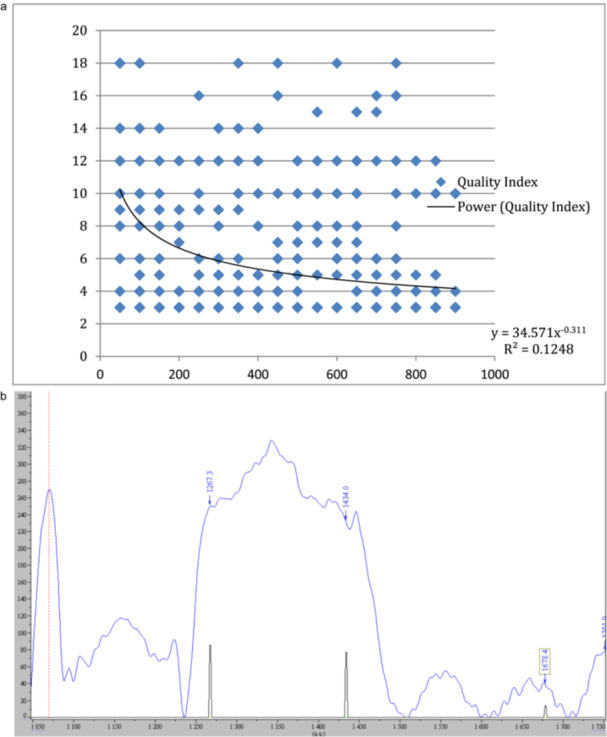
a Scatter plot of the quality of the spectra over distance (μm) from the canal. b Example of spectra dominated by background fluorescence deemed to have poor quality and data excluded from analyses.

A plot of the spectra quality against distance from the canal showed only 2–4 spectra obtained from each sampling point had QI ≤ 6 (acceptable quality) (Figure 5). The best‐fit curve displayed a steep portion near the canal lumen (at 250 μm), but its *R*‐squared value was only 0.096 with much smaller number of spectra obtained near the CDJ region. Therefore, the estimated trend should be interpreted with caution.

#### 3.4.3 Association Between Location of Sampling Site and Amide I/Amide III Band Areas Using Linear Regression (LR) Models

The integrated area of the Amide I and Amide III bands plotted against the distance from the canal lumen to the CDJ showed no visible trend of band changes. Data from control samples (T4 and T12) were analyzed using LR models incorporating Amide I or Amide III band area as the dependent variable and revealed small but significant changes in the band area over the distance from the canal lumen towards the outer root surface (Amide I: Coefficient = 8.0; 95% CI: 4.3, 11.8; Amide III: Coefficient = 7.4; 95% CI: 4.3; 10.6, respectively). The LR models including data from the test samples (T5–11) also revealed significant increase in the band areas from the canal lumen to the outer root surface (Amide I: Coefficient = 5.0; 95% CI: 4.2, 5.7; Amide III: Coefficient = 3.1; 95% CI: 2.6; 3.7, respectively), after accounting for root maturity (*p* < 0.0001), and extent of canal preparation (*p* < 0.0001). The data were subsequently stratified by tooth maturity and analyzed by a cluster of spectra every 100 μm utilizing LR models.

LR models performed using spectral data acquired within 100, 200, 300, 400, 500, 600, 700, 800, and 900 μm from the canal lumen for each root maturity group are presented in Table 4. All models, except for samples with maturity 5, revealed significant (*p* < 0.05) increase in Amide I and Amide III band areas from the lumen towards the outer root surface, regardless of the distance from the lumen or degree of root maturity but the magnitude of increase reduced towards the outer root surface (Table 4). There was a general trend showing that the magnitude (coefficient) was the largest within the 100 μm, reduced by approximately half within the 200 μm, and the magnitudes beyond 600 μm although varied, were similar to the controls (Table 4).

**Table 4:** LR models incorporating (a) Amide I, or (b) Amide III band area as the dependent variable and distance from lumen (upto 100 µm) by each degree of root maturity as the independent variable, accounting for extent of canal preparation.

		(a) Amide I		(b) Amide III	
Maturity	Distance from lumen	Coefficient	95% CI	Coefficient	95% CI
Maturity = 2 (*N* = 2 teeth, 151 sites)	Entire thickness	10.3	9.1, 11.5	6.4	5.5, 7.2
	Up to 100 µm	101.2	77.4, 125.0	70.5	47.2, 93.7
	> 100–200 µm	45.9	38.0, 53.8	30.3	23.0, 37.5
	> 200–300 µm	28.6	25.2, 32.0	19.7	15.3, 24.1
	> 300–400 µm	20.1	17.0, 23.1	13.0	10.3, 15.7
	> 400–500 µm	16.6	15.0, 18.1	10.8	8.7, 12.8
	> 500–600 µm	13.0	11.2, 14.8	7.8	7.0, 8.6
	> 600–700 µm	10.9	9.7, 12.1	6.7	5.8, 7.5
	> 700–800 µm	10.0	8.9, 11.0	5.8	5.1, 6.5
	> 800–900 µm	9.1	8.2, 10.1	5.4	5.1, 5.8
Maturity = 3 (*N* = 1 tooth, 55 sites)	Entire thickness	8.9	7.2, 10.6	6.4	5.3, 7.6
	Up to 100 µm	84.7	52.4, 119.0	52.8	31.2, 74.3
	> 100–200 µm	43.5	31.3, 55.7	26.0	18.1, 33.8
	> 200–300 µm	27.5	20.4, 34.7	18.8	16.2, 21.5
	> 300–400 µm	20.7	17.7, 23.7	12.9	10.6, 15.3
	> 400–500 µm	15.9	11.1, 20.7	13.5	5.0, 22.0
	> 500–600 µm	11.3	9.2, 13.4	8.5	5.6, 11.4
	> 600–700 µm	9.2	7.6, 10.9	7.4	5.0, 9.7
	> 700–800 µm	8.9	7.6, 10.3	6.2	4.6, 7.8
	> 800–900 µm	7.8	7.0, 8.6	5.4	4.2, 6.6
Maturity = 4 (*N* = 2 teeth, 149 sites)	Entire thickness	8.5	7.2, 9.7	5.6	4.7, 6.4
	Up to 100 µm	91.8	62.3, 121.3	59.6	42.4, 76.7
	> 100–200 µm	47.6	39.9, 55.3	26.0	21.6, 30.3
	> 200–300 µm	27.4	22.5, 32.2	16.8	14.9, 18.8
	> 300–400 µm	21.1	18.7, 23.4	14.1	9.1, 19.0
	> 400–500 µm	17.4	14.3, 20.6	12.2	10.1, 14.2
	> 500–600 µm	13.2	11.9, 14.4	8.0	6.7, 9.3
	> 600–700 µm	12.0	11.1, 12.8	7.0	5.6, 8.4
	> 700–800 µm	10.6	9.4, 11.8	6.7	4.5, 8.8
	> 800–900 µm	9.5	8.5, 10.5	6.5	4.7, 8.4
Maturity = *5* (*N* = 1 tooth, 36 sites)	Entire thickness	6.8	5.0, 8.5	4.6	3.3, 5.9
	Up to 100 µm	74.8	4.4, 145.3	59.9	−12.9, 132.7
	> 100–200 µm	34.1	−39.8, 108.0	27.7	−126.3, 181.7
	> 200–300 µm	25.0	20.0, 30.1	19.4	12.7, 26.0
	> 300–400 µm	Insufficient data	*—*	14.1	−22.5, 50.6
	> 400–500 µm	15.0	14.9, 15.2	9.1	5.0, 13.2
	> 500–600 µm	12.3	7.6, 17.0	10.2	8.4, 11.9
	> 600–700 µm	10.7	6.9, 14.0	7.0	4.4, 9.6
	> 700–800 µm	9.4	6.9, 11.9	6.4	4.0, 8.8
	> 800–900 µm	7.7	5.7, 9.7	4.4	3.3, 5.6
Maturity = 6 (*N* = 1 tooth, 75 sties)	Entire thickness	10.5	8.1, 13.0	6.1	4.6, 7.6
	Up to 100 µm	99.1	56.0, 142.1	69.9	27.5, 112.3
	> 100–200 µm	53.1	29.7, 76.6	33.0	14.8, 51.3
	> 200–300 µm	31.1	23.0, 39.3	25.1	18.2, 32.0
	> 300–400 µm	31.0	17.7, 44.3	13.8	9.8, 17.7
	> 400–500 µm	24.6	13.0, 36.3	12.4	6.5, 18.3
	> 500–600 µm	13.4	10.6, 17.0	8.9	6.9, 11.0
	> 600–700 µm	15.4	8.5, 22.3	7.3	5.3, 9.2
	> 700–800 µm	11.0	9.3, 12.6	6.3	4.4, 8.2
	> 800–900 µm	7.6	5.6, 9.6	5.4	2.0, 8.9

#### 3.4.4 Correlation Between Depth and Location of Indentation From Canal Lumen Outwards

**Figure 6:**
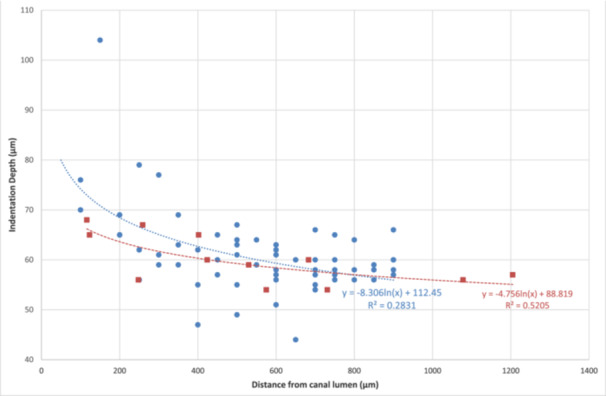
Scatter plot of depth of indentation by control (red squares) and test (blue dots) group over distance from canal lumen.

The depth of indentation (μm) on the control (T12) and test samples (T5–11) was plotted against the distance from the canal lumen (Figure 6). The indentations on the control sample (red squares) were shallower than the corresponding indentations in the test samples (blue dots). As a general trend, the indentations on the test samples were deeper near the canal lumen than those near the CDJ, particularly those within 350 μm from the lumen. The indentation depth and the distance from the canal lumen were found to follow a logarithmic trend (after testing multiple models) for both the control (*R* ^2^ = 0.52) and test (*R* ^2^ = 0.28) samples. The formulae representing the relationships are presented in Figure 6.

**Figure 7:**
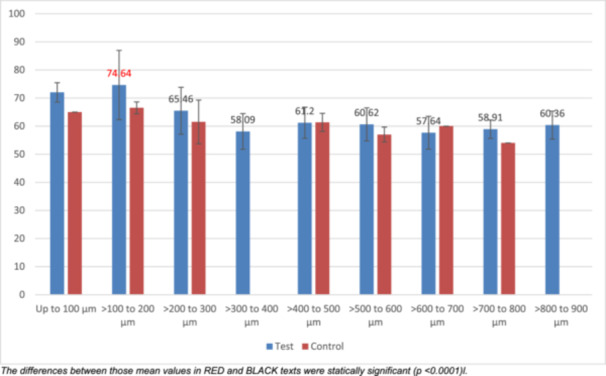
Mean depth of indentation (μm) by distance from lumen for test and control samples.

ANOVA and Bonferroni post hoc tests of pooled data from all test groups revealed the mean depth of indentations (by 100 μm intervals) reduced with distance from the canal lumen (*p* < 0.0001) with significantly (*p* < 0.05) deeper indentations within 200 μm than those further away from the lumen. The degree of root maturity only had a borderline significant effect (*p* = 0.05), whilst the extent of canal preparation did not have a significant influence (*p* = 0.2) on indentation depth. Further stratified analyses by maturity group were therefore not performed. For the control samples, there were no obvious nor significant changes in the depth of indentations (by 100 μm intervals) from the canal lumen to the CDJ (*p* = 0.3). Additionally, the depth of indentations within 200 μm of the canal lumen were significantly deeper in the test groups than the control group (*p* = 0.02) (Table 5) (Figure 7).

**Table 5:** One‐way analysis of variance comparing “depth of indentation” between “distance from lumen” by “test” and “control” groups.

Maturity	Distance from lumen	Mean	Standard deviation	*p* value for ANOVA
All test groups (*N *= 7 teeth, 123 sites)	Up to 100 µm	72.00	3.46	< 0.0001
	> 100–200 µm	74.64*	12.33	
	> 200–300 µm	65.46*	8.35	
	> 300–400 µm	58.09*	6.36	
	> 400–500 µm	61.20*	5.52	
	> 500–600 µm	60.62*	5.88	
	> 600–700 µm	57.64*	5.84	
	> 700–800 µm	58.91*	3.27	
	> 800–900 µm	60.36*	4.97	
Control group (*N* = 1 tooth, 15 sites)	Up to 100 µm	65.00	0.00	0.3
	> 100–200 µm	66.50	2.12	
	> 200–300 µm	61.50	7.78	
	> 300–400 µm	No data	—	
	> 400–500 µm	61.33	3.21	
	> 500–600 µm	57.00	2.65	
	> 600–700 µm	60.00	0.00	
	> 700–800 µm	54.00	0.00	
	> 800–900 µm	No data	—	

*Note:* The differences between those mean values highlighted with * and * were significant at the 5% level.

## 4 Discussion

The present study employed Raman spectroscopy instead of FTIR (Browne et al. 2020; Morgan et al. 2019) to determine the extent (along dentinal tubules) and boundary of NaOCl‐induced changes in dentine collagen to improve spatial resolution from 500 µm to 1 µm. In this study, the term “in‐situ mapping” refers to the spatially resolved acquisition of Raman spectra directly from intact dentine sections (along defined lines), without the need for sample pulverization or chemical extraction. Rather than employing automated spectral mapping mode, a high‐density point sampling strategy along defined transects from the canal lumen to the CDJ was adopted. This approach enabled the construction of a spatial profile of collagen degradation within the native anatomical context of the root dentine. By acquiring spectra at multiple discrete locations (up to 18 equidistant points per quadrant), it was possible to capture the gradient of chemical changes induced by NaOCl irrigation, thereby achieving effective in‐situ characterization of the extent and variability of collagen alteration, along defined lines.

The inclusion of immature and mature tooth samples facilitated investigation of the study parameters previously (Browne et al. 2020). Intact teeth were chosen to ensure that detection of altered collagen was not confounded by changes due to structural defects or caries. Availability of samples was restricted to periodontally‐involved teeth, which was considered acceptable because such involvement does not significantly impact on NaOCl‐induced changes in dentinal collagen (Browne et al. 2020). Storage in 70% ethanol with deionized water has no known effect on FTIR dentinal collagen or mineral bands (Strawn et al. 1996). Thymol solution (0.1%) was used to provide continued antimicrobial effect following initial decontamination as it does not modify Raman spectra (Soares et al. 2007).

The root canals of sample teeth were prepared to a sufficient size and taper (F3 ProTaper) to allow a degree of standardization of canal dimensions to better control the buffering capacity of the root canal system to active chlorine in the irrigant (Haapasalo et al. 2007; Sobhani et al. 2010). Silicone impressions of the root canals (T5–12) to gauge volume and extent of preparation have been judged reliable enough for the intended purpose in this study.

The adopted measure and classification for root maturity enhanced objectivity and could serve both as a discrete or continuous variable for statistical purposes. The apical portion of the root and its concomitant canal narrowing are the last features to be completed during root formation, more truly reflecting its maturity (Nanci and Cate 2008). Alternative apical size measurements (Andreasen et al. 1986; Browne et al. 2020) or tooth grouping by location of canal narrowing or root length (Rajasingham et al. 2010; Sim et al. 2001) may represent root maturity less precisely.

The higher concentration (5%) of NaOCl was chosen to ensure a measurable effect, since it is known for its influence on dentine flexural strength, modulus of elasticity, and tooth surface strain (Grigoratos et al. 2001; Rajasingham et al. 2010; Sim et al. 2001; Sobhani et al. 2010). In addition, it would allow direct comparison of findings with previous studies, utilizing the same ex‐vivo model for in‐situ localization (Browne et al. 2020; Morgan et al. 2019). Embedding the tooth in a silicone putty matrix served to simulate the apical hydrostatic resistance to irrigant penetration, as well as to protect the external root surface from the irrigant. Within its limitations, the model was representative of a clinical protocol. The duration of NaOCl residence in the canal was standardized for each tooth and followed the protocol in previous studies (Browne et al. 2020) for comparability.

Sample preparation for spectroscopy was informed by previous studies (Browne et al. 2020; Morgan et al. 2019). Tooth splitting by pre‐grooving avoided the potential risk of surface dentine compaction attributed to cutting. The flat surfaces required for both the Raman spectral analysis and indentation tests were achieved by established polishing protocols, although using 4000 grit paper would leave a smear layer (1–2 µm) (Tani and Finger 2002). However, this can be removed by surface treatment with 17% EDTA to allow more predictable FTIR analysis. (Browne et al. 2020). The protocol of 30‐sec surface treatment with 17% EDTA does not incur any substantial damage to the underlying mineralized dentine (Garberoglio and Becce 1994; Komabayashi et al. 2008).

Both Raman and FTIR spectroscopy can detect phosphate, carbonate, and Amide I and III bands, although the respective wavenumbers are different on Raman and FTIR spectra. The changes in collagen content and structure were assessed using Amide I and III bands of the Raman spectra in the present study, in contrast to Amide I and II bands employed in FTIR spectroscopy. (Browne et al. 2020; Morgan et al. 2019). These three Amide bands correspond to the main chemical groups of collagens but those that give strong IR bands may provide only weak Raman bands (Tsuda et al. 1996). The Amide II band, detected in FTIR at 1550 cm^−1^ was undetected in Raman microscopy as the intensity was too low. In this study, each Raman sampling point was visually inspected using the zoom function of the FTIR camera prior to acquisition and again before advancing to the next point. This step ensured that the laser focus was correctly aligned and that no visible damage occurred during acquisition. Despite the extended acquisition time of 24 min per point, no evidence of thermal damage, burning, or surface alteration was observed at any of the sampling sites. The laser power was maintained at a level appropriate for biological specimens, and the acquisition protocol was optimized to preserve sample integrity, while achieving adequate SNR for collagen‐specific.

To assess the spectral resolution and potential degradation, the full width at half maximum (FWHM) for the Amide I and Amide III bands was estimated from the control sample (T4). The spectra presented in Figure 2c were rescaled between 1200–1300 cm^−1^ and 1600–1700 cm^−1^ to highlight the broadening and loss of definition in these bands near the canal wall. In contrast, spectra from the control tooth (T4) (Figure 2d) exhibited consistent Amide I and III profiles across all measured distances, with no evidence of broadening or intensity loss, suggesting preserved collagen structure and no spectral damage. The calculated FWHM values for the control spectra were: 58.70 cm^−1^ for Amide I (~1667 cm^−1^); and 54.49 cm^−1^ for Amide III (~1243 cm^−1^), respectively. These values are within or slightly above the typical ranges reported in the literature, such as Xu and Wang (2012), who reported FWHM values of approximately 40–60 cm^−1^ for Amide I and 30–50 cm^−1^ for Amide III. This suggests that while the bands are broad, they remain within the expected range for hydrated dentine collagen, particularly under extended acquisition times and biological variability. The spectral quality of the control sample supports the reliability of the acquisition settings used in this study.

The acquisition time for optimal spectral quality may be affected by the type, size and protocol of sample processing, varying from seconds (for enamel) to hours or even days for dentine. Powdered dentine as sample material only requires a 60‐s acquisition period (Tsuda et al. 1996) but with the consequent loss of localization. At the study's inception, there were no published data to inform the duration of spectrum acquisition for dentine discs analyzed by Raman spectroscopy. Because of the inadequacy of spectral quality at 6 min, the duration of 12–48 min was explored. The 48‐min spectra had only a marginally higher SNR than those obtained over 24 min, leading to the adoption of the latter.

Laser irradiation of biological materials elicits fluorescence spectra from organic components that may dominate the weaker Raman signals (Tsuda and Arends 1997). The proportion of organic components and intensity of fluorescence varies in different parts of dentine. Consequently, the optimal location for Raman spectrum acquisition was first explored. Periodontally involved samples may exhibit substantial tertiary dentine deposition (Berkovitz et al. 2002) with sclerosed dentinal tubules, which may explain why intra‐tubular dentine exhibits lower fluorescence signals (Tsuda and Arends 1997).

The Amide III band represents the presence of tertiary structure in the collagen triple‐helix, while the Amide I band represents secondary structures such as the α‐helix or β‐sheet (Tronci et al. 2012). The typical position and intensity of Amide I and Amide III bands infer intact collagen fibrils with a triple helical structure, and degrees of spectral change indicate collagen structure disorganization or denaturation. Loss of the doublet infers damage to the triple helical structure of the collagen molecule.

Amide I band changes were frequently evident in their intensity (band height), which could be analyzed quantitatively. In contrast, the Amide III band changes relied on the more subjective detection of their bands’ width, the presence or absence of two bands (doublets), and the slope between the bands. Dentinal collagen change was successfully analyzed by examining the integrated area below the Amide I and Amide III bands.

Samples treated with NaOCl showed a clear reduction in absorbance bands assigned to collagen (Amide I and Amide III). The Amide I band intensity was lower in spectra obtained adjacent to the canal wall compared with spectra obtained near the CDJ, consistent with Browne et al. (2020). The Amide III band in spectra obtained closer to the CDJ exhibited a doublet, but its slope gradually flattened in spectra obtained closer to the canal walls of NaOCl‐treated samples.

Qualitative Raman analysis confirmed the loss of contour of the double slope in the Amide III band and a loss of height in the Amide I band close to the canal lumen. There was a high degree of variation among teeth and even among quadrants of the same tooth. The trend‐line of the plotted spectral quality over distance from the canal lumen was represented by a curve showing a steeply decreasing portion near the canal lumen (250 μm), followed by a more gradual reduction towards the CDJ. The poor‐quality spectra represent profound collagen degradation, in contrast to the measurable loss of contour, area under the curve, or band heights of the Amide III or Amide I bands. The plateau in the curve suggests the presence of a wide area of transition between deeply affected (≤ 250 μm) and partially affected (≥ 250μm) dentine instead of a discrete and narrow boundary effect as imagined. The findings are consistent with in situ FTIR data using an identical ex‐vivo model (Browne et al. 2020; Morgan et al. 2019) but reveal further insight into the effects beyond 500 µm.

The regression analysis presented in Figure 5 yielded a low *R*‐value (*R* = 0.1248), indicating a weak correlation between spectral quality and distance from the canal lumen. This trend was not intended to serve as a predictive model but rather to illustrate the variability in spectral quality across sampling depths. To ensure analytical robustness, spectra were rigorously assessed using a predefined quality index (QI), and only those meeting the inclusion threshold (QI ≤ 6) were retained for statistical analysis. All spectra were normalized to the carbonate band at 1072 cm^−1^, a stable and well‐characterized Raman feature of hydroxyapatite, to facilitate consistent comparison across samples. The retained data remain sufficient to support the study's conclusions regarding the spatial extent and variability of NaOCl‐induced collagen degradation in dentine.

The data suggest a significant degradation of the collagen structures beyond the secondary structure within the first 100 μm from the canal lumen, with a more irregular pattern of loss of mainly the tertiary but not the secondary structure up to 500 μm.

Comparison of the spectra from 11 locations between the canal wall and CDJ showed obvious changes in both Amide I and III bands up to 300 μm from the canal wall, but the boundary was indistinct, suggestive of progressively lesser damage at further distances. Subtle changes in collagen, reflected in the Amide I band intensity, were evident, extending the entire length up to the CDJ. The distinct damage up to 300 μm was consistent with the depth of NaOCl penetration into dentine determined using a dye (Zou et al. 2010). The boundary of NaOCl‐induced changes in dentinal collagen clearly varies by site and possibly patency of dentinal tubules (Browne et al. 2020), although the penetration into inter‐tubular dentine was impossible to determine using Raman spectra in this study.

The patency of dentinal tubules and the volume of the canal space (pre‐instrumentation) are both associated with tooth maturity, the more immature the tooth, the more patent the tubules and the wider the canal. These may, therefore, act as confounding factors and were thus quantified and analyzed. For every degree of tooth maturity (as per the index used), the Amide I and Amide III band area increased significantly between 50 μm and 100 μm from the canal lumen. Still, the magnitude of increase differed among the maturity groups with the greatest extent amongst the least mature (maturity 2) samples. Beyond this depth, there was no observable trend by maturity group. The degree of canal instrumentation had a significant favorable influence on the integrated areas of Amide I and III bands at the sampling points closest to the canal lumen. This inferred a lesser effect of NaOCl when there was a greater extent of preparation, probably explained by the removal of the less mineralized pre‐dentine layer at the canal lumen during instrumentation (Berkovitz et al. 2002), possibly coupled with generating a smear layer (combination of organic and inorganic components) acting as a protective barrier.

The Biodent indentation technique allowed every quadrant to be tested but without the same spatial resolution as the Raman analysis. The main limitation of BioDent was the lack of a microscope to aid probe positioning. Accurate measurement of the indented areas could only be performed as a post‐test image analysis of the marks left by indentation.

The outcome of the indentation test showed a non‐linear relationship between the depth of indentation and the distance from the canal lumen, with, as expected, deeper indentations near the canal lumen ( < 200 μm) and shallower values towards the CDJ, probably reflecting greater mineralization with distance away from the canal lumen. The indentations in the test group were significantly deeper than the control, proving a softening effect of NaOCl on the dentine close to the canal. In addition, there was only a borderline significant relationship between depth of indentation and index of maturity without following a particular trend, which is explainable by the variation in dentinal tubule sclerosis. There was no significant association between the depth of indentation and the degree of root canal instrumentation, which is also explainable by the reasons advanced for collagen changes related to the extent of instrumentation and smear layer formation.

The apparent contradiction in the depth of effect shown by Raman spectroscopy (approximately 500 μm) compared to BioDent (approximately 200 μm) is explained by the fact that the former is sensitive enough to detect collagen alteration that is not severe enough to induce alteration of the hardness of dentine. It is therefore not a true contradiction but simply a reflection of measurement of relatively minor changes in collagen that do not affect dentine hardness versus more severe changes that do. The relatively small distance over which this change happens is interesting probably reflecting the strong buffering capacity of dentine (Haapasalo et al. 2007). Interestingly, an ex vivo study revealed that bacterial infection extended upto 300 μm into dentinal tubules, whilst canal instrumentation using alternate 5% NaOCl and 10% EDTA rendered dentinal tubules bacteria‐free to an average depth of 130 μm from the canal lumen (Berutti et al. 1997).

Exploration of different concentrations of NaOCl and types of irrigant as well as the time‐dependent effect of NaOCl irrigation alternated with EDTA may prove helpful in this model to compare with the findings from Ramírez‐Bommer et al. (2018) on pulverized dentine, where collagen content in dentine was reduced within the first 4 min, plateauing thereafter with a conservative estimate of 16 ± 13 µm affected after 10 min. It would be also helpful to explore the extent of the effect on inter‐tubular dentine further for comparison with the effect observed on intra‐tubular dentine. Nanoindentation could be used to measure the hardness of inter‐ versus intra‐tubular dentine more precisely as the present microindentation technique could only give the average of peritubular dentine, tubule orifices and intertubular dentine (Kinney et al. 1996). Given the variability of dentinal tubules within and between teeth observed in this study, future studies could also consider increasing the sample size based on the present data for sample size estimation.

## 5 Conclusions

A Raman spectroscopy protocol was developed for in‐situ micro‐mapping of dentine collagen change along line paths, involving a 24‐min acquisition time to record adequate spectra from dentinal tubules. Raman spectroscopy and micro‐indentation successfully micro‐mapped chemical and mechanical changes in root dentine after root canal irrigation with 5% NaOCl. Root dentine collagen changes near the canal lumen (within 300–500 μm) were obvious but petered out further toward the CDJ with progressively lesser and more irregular change. The collagen changes varied amongst teeth and quadrants and with the extent of dentine removal during canal preparation. There was no discreet boundary of NaOCl effect. Micro‐hardness of root dentine reduced after exposure to NaOCl irrigation, causing the deepest indentations within 200 μm of the canal lumen. The degree of tooth maturity only had a borderline significant (*p* = 0.05) interaction with NaOCl on the depth of indentation.

## Author Contributions

**Yuan Ng:** conceptualization (equal), formal analysis (equal), methodology (equal), supervision (equal), validation (equal), visualization (equal), writing – review and editing (equal). **Michele Recchia:** conceptualization (equal), data curation (equal), formal analysis (equal), investigation (lead), methodology (equal), visualization (equal), writing – original draft preparation (lead), writing – review and editing (supporting). **Cristina Pereira:** conceptualization (equal), data curation (lead), formal analysis (equal), investigation (equal), methodology (equal), visualization (equal), writing – original draft preparation (lead), writing – review and editing (supporting). **Graham Palmer:** methodology (supporting), investigation (supporting). **Laurent Bozec:** conceptualization (equal), formal analysis (equal), methodology (equal), supervision (equal), validation (equal), visualization (equal), writing – review and editing (equal). **Kishor Gulabivala:** conceptualization (equal), methodology (equal), supervision (equal), visualization (equal), writing – review and editing (supporting).

## Funding

The authors received no specific funding for this work.

## Ethics Statement

This study was approved by the Yorkshire and The Humber—Leeds East Research Ethics Committee (REC reference number: 22/YH/0146).

## Consent

Written consent was obtained from all the participants.

## Conflicts of Interest

The authors declare no conflicts of interest.

## Supporting information

cre2.20250288‐File014.

cre2.20250288‐File015.

## Data Availability

The data that support the findings of this study are available on request from the corresponding author. The data are not publicly available due to privacy or ethical restrictions.
