# Supplementary material for: Application of Raman Spectroscopy and Micro‐Indentation to Micro‐Map the Path and Boundary of NaOCI‐Induced Dentine Collagen Changes in an Ex‐Vivo Root Canal Irrigation Model
Source: Clin Exp Dent Res. 2025 Dec 15;11(6):e70262. doi: 10.1002/cre2.70262 (PMC12705904; doi:10.1002/cre2.70262)
Supplement: Supplementary file 1 — cre2.20250288‐File014. [file CRE2-11-e70262-s001.docx]

**PRILE 2021 Flowchart**

­

**Supplementary material S1 PRILE 2021 Flowchart**
