# Supplementary material for: Application of Raman Spectroscopy and Micro‐Indentation to Micro‐Map the Path and Boundary of NaOCI‐Induced Dentine Collagen Changes in an Ex‐Vivo Root Canal Irrigation Model
Source: Clin Exp Dent Res. 2025 Dec 15;11(6):e70262. doi: 10.1002/cre2.70262 (PMC12705904; doi:10.1002/cre2.70262)
Supplement: Supplementary file 2 — cre2.20250288‐File015. [file CRE2-11-e70262-s002.docx]

Supplementary material (S2): Examples of waterfall plots of Raman spectra acquired form different test teeth (only one quadrant per tooth displayed)
